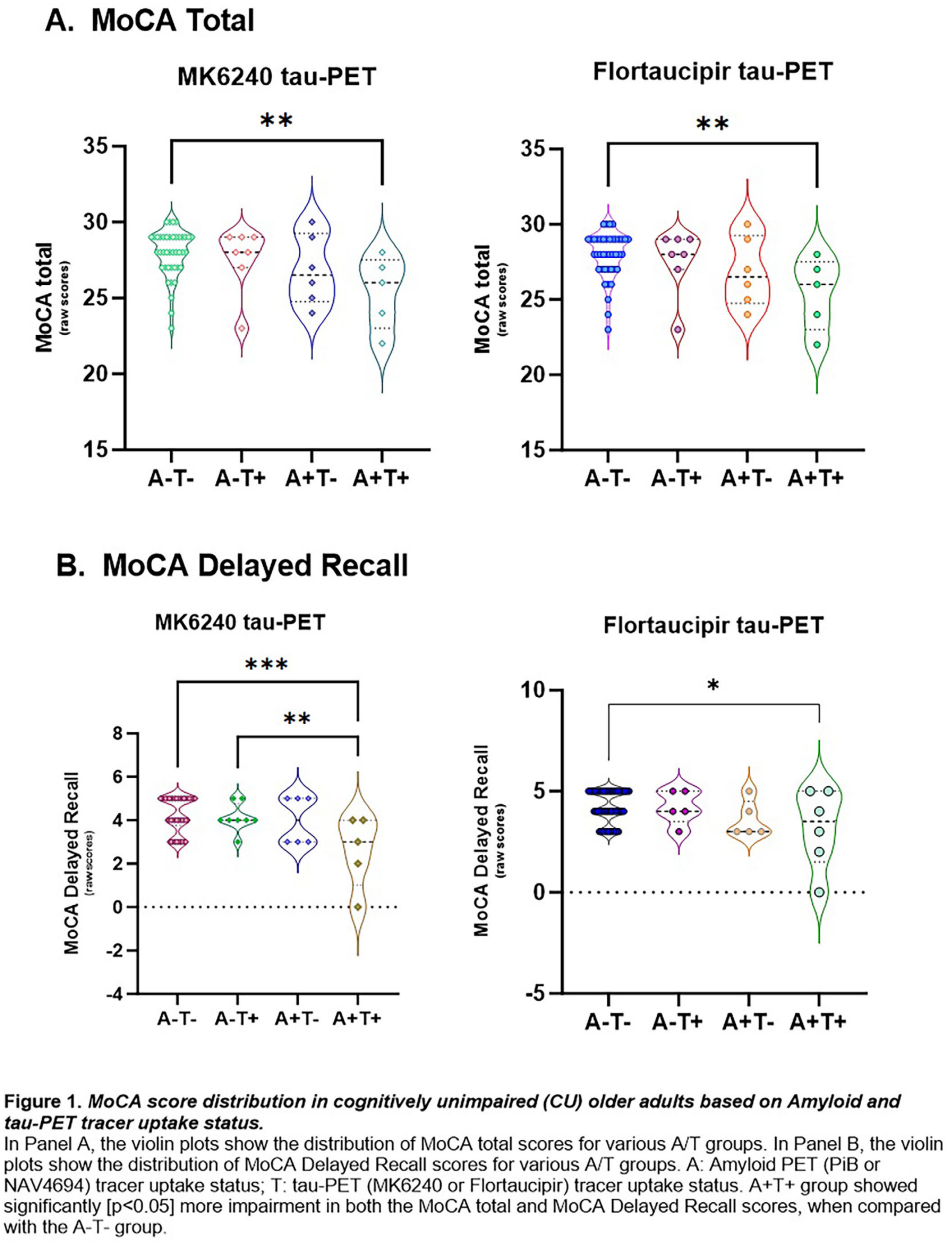# CU PET Aβ and Tau Positive Individuals Show Impairment in MoCA Delayed Recall

**DOI:** 10.1002/alz.094075

**Published:** 2025-01-09

**Authors:** Cynthia Felix, Pamela C.L. Ferreira, Matheus Scarpatto Rodrigues, Beth E. Snitz, Sarah Abbas, Markley Oliveira, Guilherme Bauer‐Negrini, Guilherme Povala, Peter Lemaire, Felix Joy Kollasserry, Marina Scop Medeiros, Carolina Soares, George Rebok, Cristiano Schaffer Aguzzoli, Bruna Bellaver, Firoza Z Lussier, Livia Amaral, Belen Pascual, Brian A. Gordon, Val J. Lowe, Hwamee Oh, David N. soleimani‐meigooni, Dana Tudorascu, William J. Jagust, William E Klunk, Pedro Rosa‐Neto, Suzanne L. Baker, Tharick A. Pascoal

**Affiliations:** ^1^ University of Pittsburgh, Pittsburgh, PA USA; ^2^ Department of Psychiatry, University of Pittsburgh School of Medicine, Pittsburgh, PA USA; ^3^ School of Medicine, University of Pittsburgh, Pittsburgh, PA USA; ^4^ Indian Statistical Institute, Bangalore, Karnataka India; ^5^ Johns Hopkins University, Baltimore, MD USA; ^6^ Global Brain Health Institute, San Francisco, CA USA; ^7^ Houston Methodist Research Institute, Houston, TX USA; ^8^ Washington University in St. Louis School of Medicine, St. Louis, MO USA; ^9^ Department of Radiology, Mayo Clinic, Rochester, MN USA; ^10^ Brown University, Providence, RI USA; ^11^ UCSF Alzheimer’s Disease Research Center, San Francisco, CA USA; ^12^ Lawrence Berkeley National Laboratory, Berkeley, CA USA; ^13^ University of Pittsburgh Alzheimer’s Disease Research Center (ADRC), Pittsburgh, PA USA; ^14^ McGill University Research Centre for Studies in Aging, Montreal, QC Canada; ^15^ Departments of Psychiatry and Neurology, University of Pittsburgh School of Medicine, Pittsburgh, PA USA

## Abstract

**Background:**

Identification of cognitively unimpaired (CU) individuals who may progress to mild cognitive impairment (MCI), is a pressing issue in the Alzheimer’s disease (AD) field, since therapeutic interventions may be more effective in the absence of cognitive impairment and neurodegeneration. CU individuals positive for amyloid and tau PET are very likely in the AD pathway. In out‐patient cognitive screening, we use rapid and simple tests such as The Montreal Cognitive Assessment (MoCA) ‐ a composite of executive, visuospatial, naming, attention, language, abstraction, delayed recall, and orientation performances. We hypothesize that sub‐categorizing the CU group based on the presence of amyloid (A) and tau (T) pathologies, as measured by PET, can reveal subtle cognitive deficits, even in a clinical test with low sensitivity such as MoCA.

**Method:**

We included 88 CU [defined as CDR global score of 0] older adults with available head‐to‐head MK6240 tau‐PET and Flortaucipir tracers from the ongoing HEAD multi‐site observational study. They also underwent Amyloid PET imaging using PiB or NAV4694. MoCA testing was done around the imaging time. To increase similarity with clinical practice, Amyloid and Tau PET positivity was defined using visual reads. Based on tracer uptake status, we categorized the individuals into A‐T‐, A‐T+, A+T‐ and A‐T‐ groups. Unpaired t‐test and one‐way ANOVA were used to test for significant differences between groups.

**Result:**

2‐tailed p‐values were significant between the A‐T‐ group and the A+T+ group for MoCA total score [p: 0.0025 and p: 0.0025]. When we stratified MoCA in the different cognitive domains, we found that the results were driven by MoCA (memory) Delayed Recall score [p: 0.0007 and p: 0.0162]. A‐T+ vs A+T+ was also significant [p: 0.0099] for MoCA Delayed Recall. Both tau tracers (MK6240 and Flortaucipir) showed similar performance for determining T+ and identifying cognitive decline.

**Conclusion:**

MoCA, a simple and commonly administered in‐office neuropsychological test, can detect subtle cognitive dysfunction in CU older adults who are Aß and tau positive (A+/T+). These CU A+/T+ individuals are likely on the path to developing AD dementia.